# Increased blood–brain barrier permeability and alterations in perivascular astrocytes and pericytes induced by intracisternal glutaric acid

**DOI:** 10.1186/2045-8118-11-15

**Published:** 2014-07-24

**Authors:** Eugenia Isasi, Luis Barbeito, Silvia Olivera-Bravo

**Affiliations:** 1 Neurobiología Celular y Molecular, IIBCE, 3318 Italia Av., Montevideo, 11600, Uruguay; 2 Institut Pasteur de Montevideo, Iguá s/n CP, Montevideo, 11400, Uruguay

**Keywords:** Blood–brain barrier, Glutaric acidemia type I, Glutaric acid, Pericyte, Astrocyte, Laminin, Neurons

## Abstract

**Background:**

Glutaric acid (GA) is a dicarboxylic acid that accumulates in millimolar concentrations in glutaric acidemia I (GA-I), an inherited neurometabolic childhood disease characterized by extensive neurodegeneration. Vascular dysfunction is a common and early pathological feature in GA-I, although the underlying mechanisms remain unknown. In the present study, we have used a previously-validated rat model of GA-I to determine the effect of GA on the blood- brain barrier (BBB) and the neurovascular unit.

**Methods:**

Newborn rat pups received a single injection of GA (1 μmol/g) or vehicle into the c*isterna magna*. BBB permeability was analyzed at 14 and 30 days post injection (DPI) by assessing Evans blue (EB) and immunoglobulin G (IgG) extravasation. Blood vessels and microglia were labeled with tomato lectin. Characterization of EB positive cells was made by double labeling with antibodies to astrocyte and neuronal markers. Immunohistochemistry against aquaporin 4 (AQP4), β receptor of the platelet derived growth factor (PDGFRβ) and laminin was used to recognize astrocyte endfeet, pericytes and basal lamina. *Zonula occludens 1* (ZO-1) and occludin striatal expression was assessed by Western blotting.

**Results:**

Perinatal intracisternal GA administration caused an increased extravasation of free EB, but not of IgG, into the striatal parenchyma at 14 and 30 DPI. EB extravasated through the BBB was internalized exclusively into neurons. GA-injected animals did not show significant changes in the area of small blood vessels in the striatum, but at 30 DPI there was a significant decrease in AQP4, PDGFRβ and laminin positive areas associated with small blood vessels. Occludin and ZO-1 expression in the striatal tissue was unchanged in all conditions analyzed.

**Conclusions:**

The present study shows a previously-unknown effect of a perinatal administration of a single intracisternal GA injection on BBB permeability and on key components of the neurovascular unit. The results suggest BBB leakage is a pathogenic mechanism and a potential therapeutic target for patients with GA-I.

## Background

Glutaric acidemia type I (GA-I) is a neurometabolic/neurodegenerative disorder caused by deficiency of the mitochondrial enzyme glutaryl-CoA dehydrogenase (GCDH) involved in the catabolism of lysine, hydroxylysine and tryptophan. Functional deficiency of GCDH leads to the accumulation of glutaric (GA), 3-hydroxyglutaric and glutaconic acids in body fluids and tissues [1-4]. Between 6 and 18 months of life, up to 90% of untreated GA-I children suffer an encephalopathic crisis, usually precipitated by infectious or viral illness. This initiates a process leading to permanent neurological deficits including dystonic-dyskinetic movement disorders, growth and cognitive impairments [2,4-6]. The pathological hallmarks of the disease include striatal and cortical degeneration, gliosis, white-matter abnormalities and vascular dysfunction [3,7,8].

Vascular pathology described in GA-I patients, include blood–brain barrier (BBB) breakdown, intradural or retinal hemorrhages, subdural effusions and chronic extravasation from trans-arachnoid vessels [3,7-10]. These pathological events may occur independently of the encephalopathic crisis [10]. Furthermore, striatal capillary distention and BBB breakdown with vasogenic oedema was observed in GCDH-deficient (Gcdh −/−) mice fed with a lysine enriched diet to boost GA production [11-13], indicating the vulnerability of the neurovascular unit to GA. However, little is known about the effect of GA on the different cellular components of the neurovascular unit. Moreover, the significance and impact of BBB dysfunction in GA-I as causative of neuronal death is still poorly understood.

The BBB is responsible for the proper functioning and homeostasis of the central nervous system (CNS) by regulating cellular and molecular trafficking between blood and brain parenchyma. Formed by specialized vascular endothelial cells, the BBB structure and function is tightly regulated by basal lamina components, pericytes, perivascular microglial cells, astrocytes and neurons which altogether constitute the neurovascular unit [14-18]. The main components of the basal lamina such as laminins interact with surface receptors from different cell types, tightly modulating the BBB [18]. Moreover, in several brain diseases, BBB breakdown causes plasma protein leakage and altered transport of molecules between blood and brain. These events are associated with neuronal death and neuroinflammation [19].

Pericytes and astrocytes have key roles in the formation and maintenance of the BBB, and in blood flow regulation and neurovascular coupling, through the release of several growth factors or via cell to cell contacts [14-18]. Pericytes are highly complex regulatory cells that are essential for the formation, maturation and maintenance of normal microvasculature [20-22], and pericyte dysfunction is associated with with several brain disorders including neurovascular diseases, neurodegeneration, multiple sclerosis and spinal cord injury [22]. However, the involvement of brain pericytes in GA-I pathogenesis is currently unknown.

On the other hand, astrocytes may play a pivotal role in GA-I pathology [23-25]. In a previously-validated model of GA-I consisting of a single intracisternal administration of a pathophysiological dose of GA to newborn rat pups reproducing an acute encephalopathic crisis similar to that described in humans with GA-I, astrocytes presented with oxidative stress, enhanced proliferation rate and S100ÃŸ overexpression [23,24]. Remarkably, this early astrocyte reactivity precedes a number of delayed pathological events such as myelination failure and neuron degeneration in the striatum [24,26]. Since astrocytes are also key cellular constituents of the BBB, we have explored whether intracisternal GA could also damage the neurovascular unit.

In the present study, using our model of GA perinatal administration [23,24,26], we determined whether a single intracisternal dose of GA was sufficient to trigger long term damage on BBB permeability and integrity by analyzing Evans blue (EB) and immunoglobulin G (IgG) extravasation and the protein expression of several components of the neurovascular unit.

## Materials and methods

### Chemicals

Evans blue, glutaric acid and all other chemicals of analytical grade were obtained from Sigma (St. Louis, MO, USA). Primary antibodies were purchased from Abcam (Cambridge, MA, USA), Invitrogen (Camarillo, CA, USA), Millipore (Billerica, MA, USA) or Sigma. Secondary antibodies conjugated to Alexa Fluor^®^ were obtained from Molecular Probes^®^Invitrogen (Eugene, OR, USA) or Thermo Scientific Pierce (Rockford, IL, USA). Material used in Western blotting assays was purchased from Bio-Rad (Hercules, CA, USA) and Thermo Scientific Pierce.

### Animals

Experimental animal work was conducted using Sprague Dawley rats, bred at the IIBCE animal house. Animals were housed in cages with food and water *ad libitum*, at controlled temperature and at 12 h light/dark cycle. Institutional guidelines according to National and International protection laws of vertebrate animals for scientific purposes were followed. All procedures were made to minimize animal pain or discomfort and were approved by the institutional IIBCE Ethical Committee. All the males from at least 5 whole litters were employed to administer GA and to perform immunohistochemistry and Western blotting assays.

### Intracisternal GA administration to rat pups

At times between 12 and 24 h after birth, each male pup was anesthetized with hypothermia by placing in an aluminum foil-lined tube packed in crushed ice for10 min until most reflexes were absent. Then, each animal was injected into the c*isterna magna* with either GA (1 μmol/g body weight, pH 7.4) [3-5], or an equal volume of vehicle (phosphate buffered saline (PBS), 10 mM, pH 7.4). A maximal volume of 5 μl was injected using a 30G needle attached to a Tygon tube extension to allow correct manipulation and zone identification [23,24,26]. The amount of GA administered ensured immediate high brain concentrations (~100 mM in the cerebrospinal fluid); which is hard to obtain by systemic injection due to limited flux across the BBB [27]. After injection, all animals were allowed to recover at 30°C on a heating pad for 30 min and then returned to their mothers until processing.

### Animal perfusion with a paraformaldehyde-EB mixture

EB, a diazo blue dye of 960 Da that becomes fluorescent when attached to proteins, only leaks to the brain parenchyma when BBB is absent, immature or significantly disrupted, thus, it is widely used to assess BBB integrity [28]. EB extravasation studies were performed following the method proposed by del Valle *et al.*, with minor modifications [28]. At 14 and 30 days post-injection (DPI), GA and vehicle-injected rats (controls) were anaesthetized with 90:10 mg/Kg ketamine/xylazine and then intracardially perfused with a volume of saline solution followed by a mixture of 1% EB in 4% fresh paraformaldehyde (PFA) in 100 mM PBS (pH = 7.4). After that, the brains were dissected, maintained overnight in 4% PFA at 4°C and kept in PBS-0.05% sodium azide at 4°C, until sectioning.

### Analysis of EB extravasation by light and fluorescence microscopy

Using a 1000S Leica vibratome, 50 μm thick consecutive coronal sections, covering the whole striatal region, were obtained and stored free-floating at 4°C in PBS with 0.05% sodium azide. Light microscopy images and far red fluorescence EB emission was detected by epifluorescence or confocal microscopy with a 633 nm HeNe laser attached to an Olympus FV300 laser confocal microscope. EB emission capture was performed either alone or after immunostaining with different markers. Images from age-matched animals injected with vehicle or GA were taken with the same acquisition settings (same excitation/emission and exposure parameters). Quantitation of the EB red fluorescence emission, measured as the intensity per area unit (mean gray value: averaged intensity per pixel), was done in the whole striatum and parietal cortex of vehicle- and GA-injected animals by using the Image J software (NIH, USA). Values represented are the means ± S.E.M., determined after analyzing the raw data from each experimental condition with the statistical descriptive tool of Sigma Stat2.0 program.

### IgG recognition in striatal tissue

Brain sections containing the striatum from 3 animals of each experimental condition were permeabilized with 0.1% Triton X-100, blocked with 5% BSA and then incubated for 2 h at room temperature (RT) with 1:300 dilution of an anti-rat IgG conjugated to FITC (Pierce). After that, sections were washed 3 times with PBS, mounted in 1:1 glycerol-PBS containing 1 μg/ml Hoechst 33342 and imaged in a FV300 Olympus confocal microscope provided with 405, 488 and 633 nm lasers. IgG fluorescence was analyzed by using the 488 nm laser and corresponding filters. As negative controls the rat IgG antibody was omitted.

### Lectin histochemistry

Striatal sections from vehicle- and GA-injected animals at 14 and 30 DPI were permeabilized with 1% Triton X-100 for 20 min at RT, quickly rinsed and incubated overnight with 5 μg/ml of *Lycopersicon esculentum* biotinylated lectin (Sigma). After 3 washes with PBS, sections were incubated with a 1:500 dilution of Alexa 488 streptavidin (Molecular Probes^®^). After 90 min, sections were washed 3 times, mounted in 50% glycerol and imaged in a confocal microscope. Green positive areas of blood vessels were quantified for both experimental conditions after manually delineating all vessel profiles <10 μm in diameter in a field of 211.5 mm^2^. At least 5–7 fields per section, 3–5 sections per animal and 3–5 animals per condition were analyzed.

### Basic histology

At least 3 sections of each experimental condition were permeabilized with 0.3% Triton X-100 for 20 min and then incubated with 0.05% Cresyl Violet (Sigma) for 2 min at RT. Sections were progressively dehydrated with increasing ethanol concentrations, acetone and xylene and finally mounted with DPX (Fluka). Microphotographs of the striatal regions were obtained in an IX81 Olympus microscope (Center Valley, PA, USA).

### Immunohistochemistry

For each animal and staining procedure, 5 equivalent sections covering the anterior and medial striatum (roughly between 2.28 and 0 mm to Bregma, according to Paxinos and Watson [29] were analyzed. Anatomical landmarks were used to ensure that parameters were analyzed at similar levels within and between groups. Photomicrographs of representative areas were taken under similar conditions of acquisition to controls. Immunohistochemistry assays were performed on free-floating sections. Sections were washed with PBS; permeabilized 20 min with 0.05% Triton X-100 and incubated 30 min in blocking buffer (PBS containing 0.05% Triton X-100 and 5% bovine serum albumin (BSA)). Afterwards, sections were incubated with one of the following antibodies: anti-S100β (1:400, Sigma), anti-laminin 1 + 2 (1:1000, Abcam), anti-aquaporin 4 (AQP4, 1:75, Millipore), anti-β receptor of the platelet derived growth factor (PDGFRβ, 1:100, Abcam), anti- *zonnula occludens* 1 (ZO-1, 1:200, Invitrogen), or anti-neuronal nuclear protein (NeuN, 1:200, Millipore). All dilutions were made in PBS containing 0.05% Triton X-100. After overnight incubation at 4°C, sections were rinsed in PBS, and incubated at room temperature (RT) for 90 min with a 1:500 dilution of 1 mg/ml corresponding secondary antibodies conjugated to Alexa Fluor^®^ 488 (Molecular Probes^®^). Sections were then washed, mounted in 1:1 glycerol-PBS containing 1 μg/ml Hoechst 33342 and imaged in a FV300 Olympus confocal microscope. As negative controls the primary or secondary antibodies were omitted. Quantitation of positive areas was done by using the Image J software in manually delineated blood vessels that have a diameter less than 10 μm. Positive areas measured in similar striatal surfaces from each brain section were averaged to obtain one value per animal. Data represented are the mean ± S.E.M. of the values obtained in 3–5 animals per condition.

### Western blotting

To assess the expression of occludin and ZO-1 in the striatal tissue of GA or vehicle-injected animals, 3 different batches of striatal samples were obtained from 14 and 30 DPI animals and then processed for Western blotting analysis. Briefly, fresh samples were collected in tissue lysis buffer, homogenized and protein quantified with the bicinchoninic acid method. Denatured samples were seeded and a typical SDS-PAGE electrophoresis and blotting was performed [30]. Proteins that were transferred to a PVDF membrane were further incubated overnight at 4°C with a 1:1000 dilution of anti-occludin (abcam) or anti-ZO-1 (Invitrogen) antibodies. 1:4000 of anti-βactin antibody (Sigma) was used as a protein loading control. After 1 h of incubation at RT with peroxidase-conjugated secondary antibodies (Bio-Rad), the reaction product was developed with the Pierce ECL kit and bands were analyzed with the Image J gel analyzer tool. Data were expressed as the percentage of β-actin expression in each corresponding sample.

### Statistical analysis

Data analysis was performed with Sigma Stat 2.0 or Graphpad Prism 3.0 using the student *t*-test in parametric populations. All data was expressed as percentage of respective age-matched controls and was obtained from at least 3–5 independent experiments. Results presented are the means ± SEM and p < 0.05 was considered statistically significant.

## Results

### Intracisternal GA perinatal administration causes increased EB extravasation in the striatum

**Figure 1:**
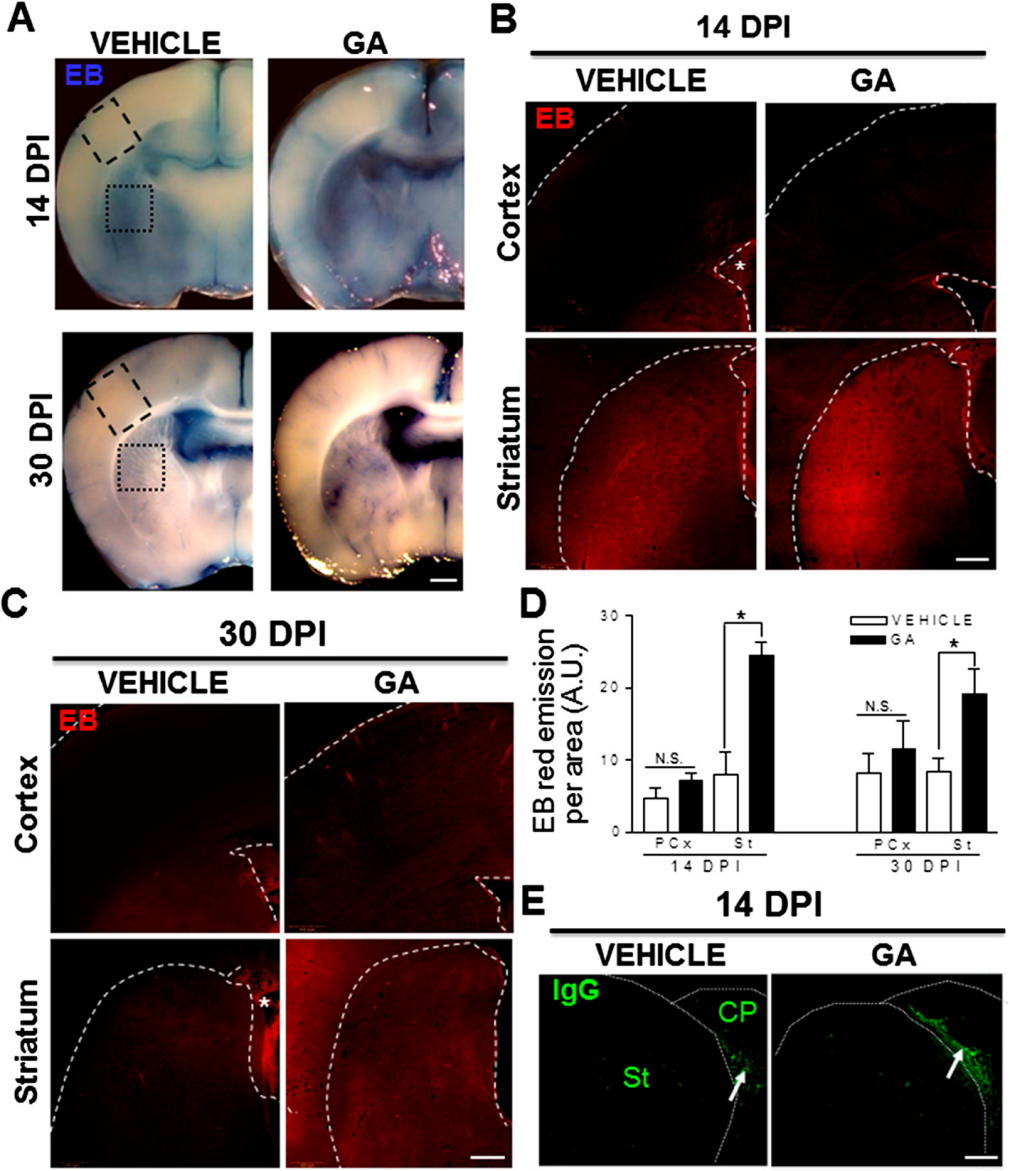
**Increased permeability of the BBB after a perinatal intracisternal injection of GA. (A)** Panoramic light microscopy images of EB staining (blue) in brain coronal sections in vehicle- and GA-injected animals at 14 and 30 DPI. Note the more intense staining in GA-injected animals, mostly in the striatal region, indicating increased BBB permeability. **(B-C)** Epifluorescence images of EB red emission in the parietal cortex and striatum at levels indicated in the dashed line boxes shown in **(A)**. Increased parenchymal EB staining was found in GA-injected animals when compared to controls. It was also evident that zones that normally do not have a BBB (*) were stained in all animals perfused with EB. Some EB parenchymal extravasation was found in vehicle-injected animals at 14 DPI, when the BBB is not fully established. **(D)** Quantitation of EB red fluorescence in the parietal cortex (PCx) and striatum (St) indicating a significant increase in the striatum of GA-injected animals at both 14 and 30 DPI when compared to controls. (*): p < 0.05. **(E)** Confocal images of IgG immunostaining (green) in the striatum of 14 DPI vehicle- and GA-injected animals. Note that the positive signal is present only in the choroid plexus (CP, white arrows) but not in the striatum. Scale bars in A: 1 mm; B; C and D: 500 μm; E: 200 μm.

Rat pups were injected at postnatal day 0 with PBS or GA into the *cisterna magna* to experimentally reproduce a GA-I like encephalopatic crisis as previously reported [23,24,26]. Then, animals were perfused with 1% EB in 4% PFA at 14 or 30 DPI to analyze EB extravasation. EB staining was located in blood vessel lumen or wall, in the lateral ventricles and in the parenchyma of areas lacking a BBB such as the circumventricular organs, in all experimental conditions (Figure 1). At 14 DPI, light microscopy images of coronal sections evidenced striatal regions stained with blue in control animals but there were more extensive and more strongly-stained regions in GA-injected animals. Accordingly, EB red fluorescence was increased mostly in the striatum (Figure 1A, B). At 30 DPI, control animals showed EB extravasation restricted to the brain areas that lack BBB with an almost undetectable blue and red staining in cortical and striatal parenchyma. In contrast, a significant increase in EB extravasation was seen in GA-injected animals (Figure 1A, C) and compared to controls, there was a 3-fold and ~2.5- fold increase in total EB red fluorescence in the striatum of GA-injected animals at 14 and 30 DPI, respectively (Figure 1D). In contrast, IgGs were not decteted in the brain parenchyma of GA-injected animals, but were only recognized in the choroid plexus from both vehicle and GA-injected animals at 14 DPI (Figure 1E) and 30 DPI (data not shown). This suggests that the BBB leakage in GA-injected animals is restricted to low MW molecules.

### EB labeled striatal neurons in GA-injected animals

**Figure 2:**
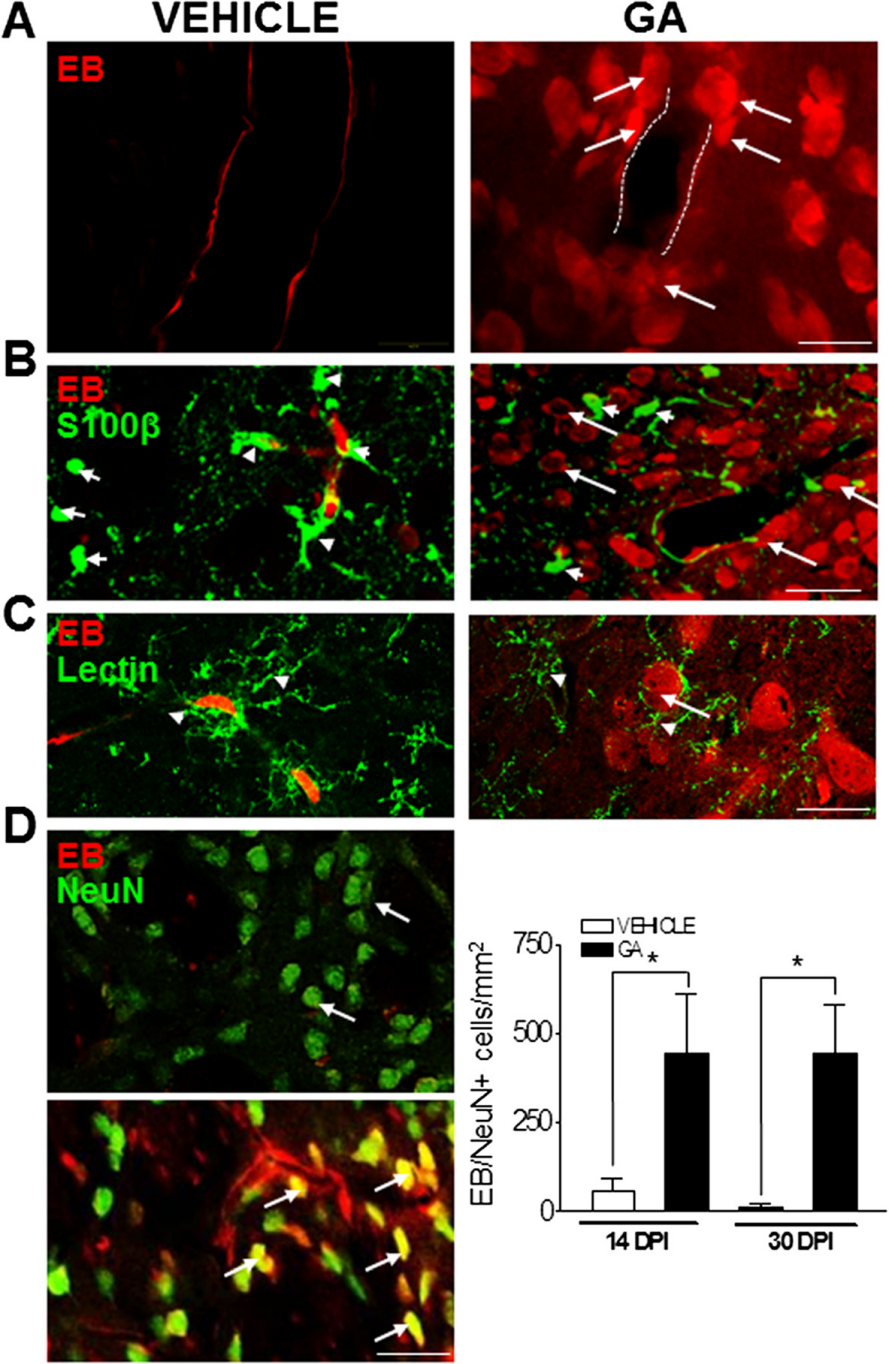
**EB labels striatal neurons upon GA perinatal administration. (A)** Confocal images of the striatum evidencing that in GA-injected animals EB leaked into the parenchyma and entered many cells that surrounded the blood vessel (white dashed lines) and have a typical neuronal morphology (white arrows). In contrast, in vehicle-injected animals EB was restricted to the blood vessel wall. **(B)** EB did not permeate the cell membrane of S100β positive astrocytes in GA-injected animals (green, short white arrows) either in the striatal parenchyma or in the processes close to blood vessels, but it stained numerous rounded cells (long white arrows) that resembled the morphology of striatal neurons. **(C)** Lectin-positive microglia (arrowheads) were not labeled with EB. It was also evident that microglia are in close contact with EB positive neuron-like (white arrow) cells in GA-injected animals. **(D)** Co-localization of EB (red) and NeuN (green) was observed in many striatal neurons (yellow, white arrows, lower picture) in GA-injected animals. In contrast, the striatum from vehicle-injected animals did not show co-localization and green NeuN positive cells were typically arranged (upper picture). The quantitation of double labeled EB/NeuN neurons evidences a significant increase (* at p < 0.05) both at 14 and 30 DPI, with respect to controls. Scale bars in A and C: 15 μm B and D: 50 μm.

Because EB can easily attach to proteins and become fluorescent, confocal analysis of EB fluorescence allowed the identification of EB positive cells surrounding blood vessels in GA-injected animals (Figure 2A). EB did not co-localize with S100β-positive astrocytes that were found widespread throughout the whole striatal parenchyma with processes surrounding the blood vessels (Figure 2B), neither with tomato lectin-positive microglial cells [31] that were found dispersed throughout the striatal parenchyma and around EB positive cells in GA-injected animals (Figure 2C). Conversely, most of the EB positive cells displayed a typical morphology of striatal neurons and co-labeled with the pan-neuronal marker NeuN, and the number of double-labeled EB/NeuN positive neurons in the striatum of GA-injected rats was 8 and 40- fold higher than controls at 14 and 30 DPI, respectively (Figure 2D). Hence, all data suggest that EB was internalized exclusively into neurons.

### Decreased expression of astrocyte endfeet and pericyte markers around blood vessels after GA administration

**Figure 3:**
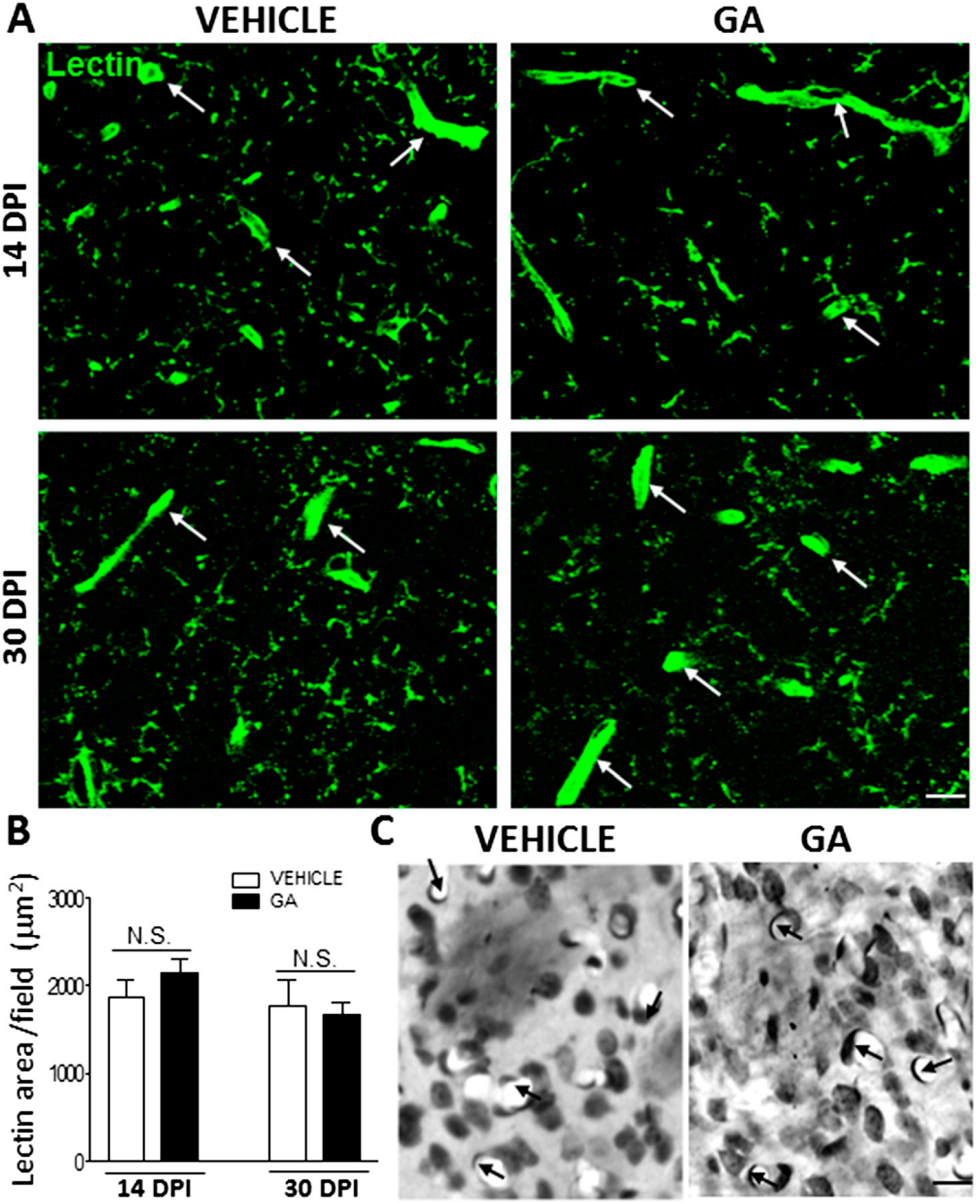
**Brain microvessel area was not affected by intracisternal GA injection. (A)** Confocal images of blood vessel profiles (white arrows) and microglia processes positive to lectin in both vehicle- and GA-injected animals at 14 and 30 DPI. **(B)** Quantitation of lectin-positive areas per field in manually-delineated small vessels (<10 μm in diameter) showing no significant differences in GA-injected animals with respect to controls. **(C)** Cresyl Violet staining of striatal sections evidencing the preservation of small blood vessel (black arrows) number and appearance. Note flattened endothelial cells lining the internal wall of capillaries in both vehicle- and GA-injected animals. Scale bars in **A** and **C**: 15 μm.

**Figure 4:**
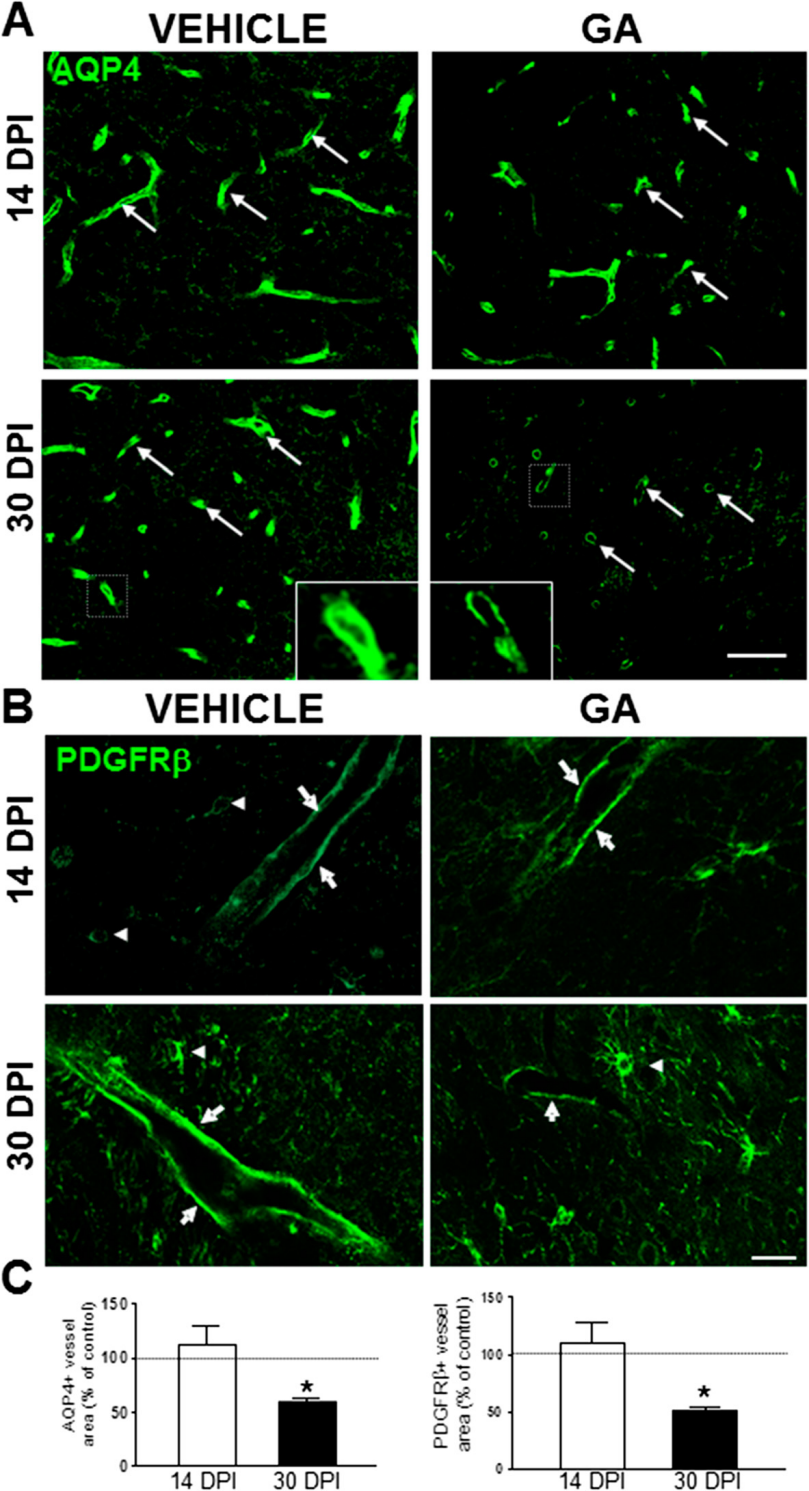
**GA perinatal administration affected astrocytes and pericytes. (A)** Immunofluorescence images of AQP4 signal delineating blood vessels (white arrows) in the striatum of vehicle and GA-injected animals. No significant changes were observed at 14 DPI. At 30 DPI, AQP4 became a tiny ring around some vessels in GA-injected animals, as clearly shown in the higher magnifications of the areas enclosed. **(B)** Representative images of the immunoreactivity for the pericyte marker PDGFRβ evidencing strong signal around some blood vessels (white arrows) and some middle-sized positive cells (arrowheads). At 30 DPI, there was a reduction in the PDGFRβ signal around blood vessels of GA-injected animals when compared to controls. **(C)** Quantitation of AQP4 (left) and PDGFRβ (right) immunoreactivity confirming a significant decrease in the positive areas surrounding blood vessels in GA-injected animals related to age-matched controls at 30 DPI. Values are represented as the percentage of age-matched controls. (*) indicates statistical significance at p < 0.05. Scale bars in A: 50 μm and C: 20 μm.

Tomato lectin histochemistry was employed to assess the effects elicited by perinatal GA administration on the striatal microvasculature density. Besides staining microglial cells recognized by its delicate pattern of positive thin processes, lectin also bound to the endothelial glycocalyx allowing visualization around 40 profiles of small blood vessels (<10 μm) per field in both control and GA-injected animals (Figure 3A). Quantitative analysis of lectin-positive small vessel area, indicated that perinatal intracisternal GA did not affect the total surface area of the striatal microvasculature at 14 and 30 DPI (Figure 3B). Lectin staining did not identify any significant effect on microvessel morphology or number. This was confirmed by the staining of striatal sections with cresyl violet which showed the typical appearance of flattened endothelial cells lining the lumen of small vessels in both vehicle- and GA-injected animals (Figure 3C).Recognition of astrocyte endfeet by AQP4 immunoreactivity showed clear blood vessel profiles in vehicle-injected animals at both 14 and 30 DPI (Figure 4A). In GA-injected animals, the signal decreased until it became a weak thin rim bordering some vessels in cross section at 30 DPI. At that time, the reduction of AQP4 positive areas that surrounded blood vessels was ~50% compared to controls (Figure 4C). On the other hand, the immunoreactivity for the pericyte marker, PDGFRβ, showed a strong signal delineating some blood vessels and staining pericytes which appeared as small round cells with smooth tiny processes located close to blood vessels in control animals (Figure 4B). In GA-injected animals there were no changes at 14 DPI with respect to controls, but at 30 DPI, the strong signal around blood vessels was decreased and the total PDGFRβ + area was reduced by 50% (Figure 4C). Some PDGFRβ-positive cells remained dispersed throughout the striatal parenchyma possibly evidencing a pericyte detachment from blood vessels that needs further confirmation.

### GA effects on laminin, ZO-1 and occludin expression

**Figure 5:**
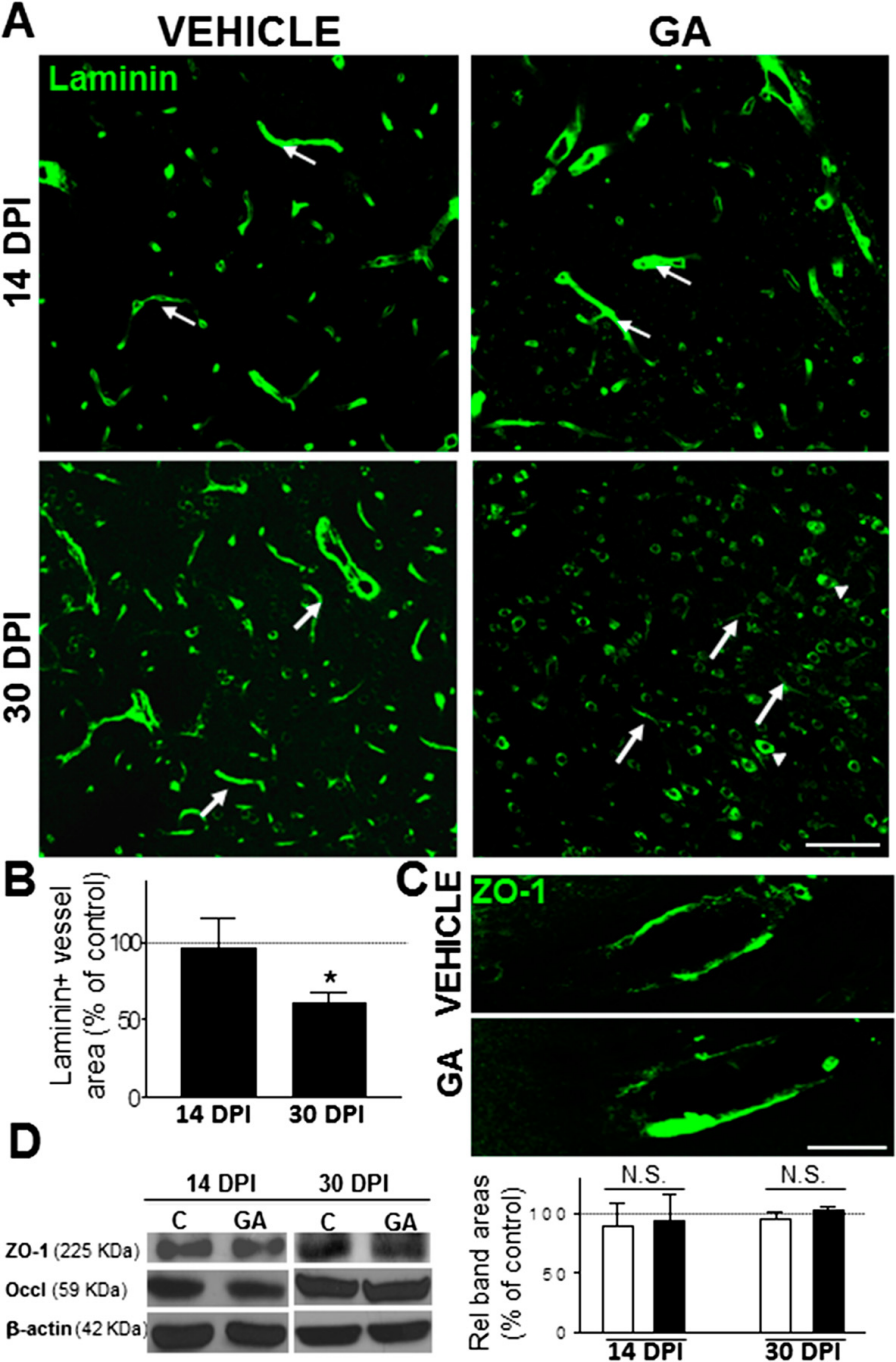
**Reduced laminin expression around small blood vessels in the striatum of GA-injected animals. (A)** Representative images of brain sections at the striatal level showing laminin immunoreactivity that delineates blood vessel profiles (white arrows) in control animals. In GA-injected animals laminin expression seemed unaffected at 14 DPI but an important reduction was found at 30 DPI, together with a simultaneous increased expression of cytoplasmic laminin staining in neuronal-like cells (arrowheads). **(B)** Quantitation of the laminin-positive area surrounding manually-delimited blood vessels <10 μm in diameter showed a statistically-significant decrease at 30 DPI in GA-injected animals. Data represented is the percentage of respective controls. **(C)** High magnification images of ZO-1 immunoreactivity in striatal vessels of vehicle- and GA-injected animals at 30 DPI evidencing absence of changes in the distribution and intensity of the signal. Scale bars in A: 50 μm and C: 15 μm. **(D)** Western blotting images (left panel) and quantitation of the endothelial tight junction proteins, occludin and ZO-1 (right panel). β-actin was used as a protein loading control. Data represented are the percentage of areas related to controls that were indicated as 100% in the chart (dashed black line). Neither occludin nor ZO-1 changed its overall expression in the striatal tissue of vehicle and GA-injected animals, at both 14 and 30 DPI.

The confocal analysis of laminin, an extracellular glycoprotein enriched in basement membranes associated with blood vessels [18], revealed a significant (40%) reduction in positive areas surrounding small vessels (<10 um in diameter), in GA-injected rats at 30 DPI when compared to controls (Figure 5A, B). Moreover, intracellular laminin staining was found in all animals, but was increased in GA-injected rats (Figure 5A). On the other hand, the expression levels of endothelial cell tight junction proteins, ZO-1 and occludin, were not modified in striatal tissue of GA-injected rats (Figure 5C, D). ZO-1 immunohistochemistry revealed a preserved localization of the signal in the striatal vasculature (Figure 5C).

## Discussion

Because intracisternal GA administration to rat pups is sufficient to elicit an encephalopathic-like crisis and subsequent progressive glial activation and neuronal death in the striatum [23,24,26], we have analyzed the effect of a single GA perinatal injection on BBB permeability and structural components to know if this can contribute to GA-I pathogenesis. Here we showed that intracisternal GA administration to newborn rats resulted in long term striatal BBB leakage and reduced expression in some markers of the neurovascular unit. Analysis of EB extravasation and IgG immunoreactivity in rats injected with GA indicated a significant increase in BBB permeability restricted to the lower MW marker, in the striatum at 14 DPI and 30 DPI, when the normal BBB is totally mature and the transendothelial electrical resistance reaches its maximum value [32,33]. Hence, these results strongly suggest an abnormal long-term leakage of the BBB upon GA exposure and are in accordance with the increased BBB permeability observed in Gcdh −/− mice subjected to dietary high lysine supplementation [11,13], a condition that boosts the endogenous production of GA-I metabolites in the brain.

Remarkably, leakage of EB into the parenchyma allowed the visualization of cells stained with EB in the striatum of GA-injected animals. EB co-localized with the neuronal marker NeuN, but not with either astrocytes or microglial cells recognized by S100β immunoreactivity or tomato lectin, respectively. Although we cannot ignore that EB could label some healthy neurons, this result suggests that EB labels a subset of neurons in which plasma membranes are altered sufficiently to allow dye to enter [28,34-36]. Since, Olivera *et al.*[24] previously demonstrated that GA-injected animals have significant striatal neuronal death at 21 and 45 days after GA administration, our present data indicate that GA-induced defects on BBB permeability precede neuronal damage.

Intracisternal GA did not produce any significant effect on the density of lectin-positive small blood vessels in the striatal parenchyma. However, it caused a delayed reduction in some markers of the neurovascular unit, such as PDGFRβ, AQP4 and laminin associated with small blood vessels, indicating an indirect GA response. At 30 DPI, the immunoreactivity of the pericyte marker PDGFRβ associated with small striatal blood vessels was strongly reduced, suggesting an indirect vulnerability of pericytes to GA. Pericyte malfunction has been associated with a dysfunctional BBB as well as with capillary dilation and impaired blood flow control [22,37]. Thus, the involvement of pericytes may explain, at least in part, altered cerebral hemodynamics with reduced blood flow in GA-I patients [9]. Similarly, possible GA-induced changes in pericyte density, localization and/or function may contribute to the microvascular abnormalities and BBB defects reported in the disease, as has been described in different pathological conditions, including trauma, diabetic retinopathy, stroke and LPS-induced sepsis [38,39].

The decreased AQP4 immunoreactivity in GA-injected animals suggests structural and possibly functional defects in the astrocytic endfeet associated with the microvasculature. AQP4 clustering in astrocytic endfeet contributes to water homeostasis and spatial potassium buffering, playing a pivotal role in the neurovascular coupling. While polarized AQP4 expression is lost in many conditions of acute or chronic brain injury [16,40,41], this is the first report suggesting the involvement of AQP4 in GA-I. Since GA induces an early and strong striatal astrocyte reactivity evidenced by enhanced proliferation and S100β overexpression [23,24,26], AQP4 downregulation might be a consequence of a progressive altered differentiation and polarization of astrocytes at the neurovascular unit that became apparent several weeks after GA injection.

Reduced laminin expression around small blood vessels further supports a microvascular compromise induced by GA. In a previous study, ablation or acute disruption of astrocytic laminin altered astrocyte endfeet organization, leading to hemorrhages in deep brain regions and impaired function of vascular smooth muscle cells [42]. Furthermore, the clustered distribution of AQP4 in astrocytes appeared to be mediated by interaction with extracellular laminin-1 [43]. Similarly deletion of β2 and β3 isoforms of laminin caused a reduction in AQP4 expression and function in the Müller glia [44]. Taken together, our results suggest that perinatal GA administration significantly affects the establishment of the neurovascular unit during postnatal life.

On the other hand, GA did not induce significant effects on the expression of two major components of endothelial tight junctions such as ZO-1 and occludin [18]. Previous results in the Gcdh−/− mice fed with a high lysine diet showed BBB defects evidenced by a progressive disappearance of occludin with no changes in ZO-1 expression [13]. Although we have not demonstrated GA effects on tight junction organization and ultrastructure, as has been shown for other brain diseases [16], our present results suggest that EB extravasation through a disrupted BBB would not necessarily require a decomposition of endothelial tight junctions. Accordingly, increased transendothelial vesicle trafficking could be a plausible mechanism that may explain BBB leakage when tight junctions remain unchanged, as was reported in a model of embolic stroke in rats [45].

Although we did not analyze the primary cellular targets of GA that account for the BBB damage, previous results strongly suggest that astrocytes may be the earliest GA targets as they would be able to internalize this acid [46,47], thus altering their function and signaling capabilities [23-26]. It is possible that early astrocyte dysfunction elicited by GA including S100β overexpression [23,26] and possibly altered ammonia clearance causing hyperammonemia as reported in Jafari *et al.*[25], may account for the increased BBB permeability [48,49], at least early after GA administration. Moreover, the persistence of astrocyte-enhanced proliferation and immaturity [23,24] several weeks after GA-injection may further sustain a cascade of pathological events responsible for BBB disruption and altered expression of key components of the neurovascular unit. This hypothesis seems to be consistent with the fact that a brief and transient increase in GA brain concentration during the early postnatal period is enough to trigger long lasting deleterious effects on BBB function and cellular markers and subsequent delayed neuronal death. Most of the pathological events triggered by a single exposure to GA are delayed by several weeks, implying an active and autonomous “toxic” process that is maintained independent on GA levels.

## Conclusions

We have shown that a single intracisternal GA perinatal administration elicited a previously unknown effect on the neurovascular unit leading to a sustained hyperpermeability towards low MW Evans blue dye. This suggests that disruption of the BBB is a pathogenic mechanism and a potential therapeutic target in GA-I. Further studies must be addressed to establish the mechanisms underlying this phenomenon and their relationship to GA-I progression and outcome.

## Abbreviations

AQP4: Aquaporin 4; BBB: Blood–brain barrier; DPI: Days post-injection; EB: Evans Blue; GA: Glutaric acid; GA-I: Glutaric acidemia type I; GCDH: Glutaryl-CoA dehydrogenase; IgG: Immunoglobulin G; NeuN: Neuronal nuclear protein; PDGFRβ: Receptor β of the platelet derived growth factor; ZO-1: Zonnula occludens 1.

## Competing interests

The authors declare that they have no competing interests.

## Authors’ contributions

EI and SOB designed the experiments. EI performed most of the experiments, SOB participated in some experiments. EI, SOB, LB wrote the manuscript. All authors read and approved the final manuscript.
